# Hemophagocytic Lymphohistiocytosis Secondary to Visceral Leishmaniasis: A Case Report

**DOI:** 10.7759/cureus.75360

**Published:** 2024-12-09

**Authors:** Joana Subtil, Rui Carvalho, Renata Silva, Ana Filipa Rebelo, Fernando Guimarães

**Affiliations:** 1 Internal Medicine, Centro Hospitalar de Trás-os-Montes e Alto Douro, Vila Real, PRT

**Keywords:** hemophagocytic lymphohistiocytosis (hlh), human immunodeficiency virus (hiv) infection, leishmania amastigotes, pneumocystis jiroveci pneumonia, visceral leishmaniasis (vl)

## Abstract

Hemophagocytic lymphohistiocytosis (HLH) is a rare clinical entity characterized by fever, constitutional symptoms, and hepatosplenomegaly associated with the presence of hemophagocytosis in the bone marrow and other organs. Visceral leishmaniasis (VL) is a severe zoonotic disease hypoendemic in Portugal, particularly in the Alto Douro region.

We report the case of a 21-year-old female patient with a recent diagnosis of human immunodeficiency virus (HIV) infection, stage C3, in the context of severe *Pneumocystis jirovecii* pneumonia, who presented to the emergency department with fever, erythematous rash on the upper limbs and trunk, choluria and jaundice, one week after starting antiretroviral therapy (ART). On admission to the emergency department, she was febrile and tachycardic, but hemodynamically stable. Blood tests showed pancytopenia and slight cholestasis. She was diagnosed with toxic hepatitis and hematologic toxicity secondary to ART and cotrimoxazole, and both therapies were discontinued, switching prophylaxis to atovaquone. During hospitalization, she maintained a fever despite empirical antibiotic therapy with piperacillin/tazobactam and worsening pancytopenia. Microbiological tests were negative. Blood work revealed an elevated ferritin and triglycerides. Presenting multiple criteria for hemophagocytic lymphohistiocytosis, she was submitted to a bone marrow aspirate, showing *Leishmania amastigotes* and aspects of hemophagocytosis. Diagnosed with HLH secondary to VL, she received treatment with liposomal amphotericin B, with clinical and analytical improvement.

Given the rarity of this entity, its diagnosis and treatment can represent real challenges in clinical practice. Early diagnosis reduces morbidity and mortality.

## Introduction

Hemophagocytic lymphohistiocytosis (HLН) is an aggressive and life-threatening syndrome characterized by excessive immune activation and tissue infiltration by activated macrophages and lymphocytes, leading to severe inflammation and multi-organ dysfunction.

The etiology of HLH can be broadly categorized into primary (familial) HLH, more common in the pediatric population, and secondary (sporadic) HLH, affecting all age groups. While primary HLH is typically caused by inherited genetic mutations that impair the cytotoxic function of natural killer (NK) cells and CD8+ T cells, secondary HLH can be triggered in the context of infections, malignancies, autoimmune diseases, or other immune dysregulation conditions. Common infectious triggers include Epstein-Barr virus (EBV) and cytomegalovirus (CMV) [1-3].

The diagnosis of HLH involves a combination of clinical, laboratory, and histopathological criteria. The HLH-2004 diagnostic criteria, established by the Histiocyte Society, are widely used though their sensitivity and specificity remain unknown [4]. These criteria include fever ≥38.3°C; splenomegaly; cytopenias (affecting at least two of three lineages in the peripheral blood); hypertriglyceridemia (>265 mg/dL); hemophagocytosis in bone marrow, spleen, lymph nodes or liver; low or absent NK-cell activity; ferritin >500 ng/mL; soluble CD25 ≥2400U/mL [4].

Visceral leishmaniasis (VL), caused by protozoan parasites of the leishmania species, is transmitted by infected sandflies. In Portugal, it is relatively rare but remains a significant public health concern, particularly in the Mediterranean region where it is hypoendemic [5].

VL is a rare trigger for HLH that usually occurs in immunosuppressed patients. VL and HLH have overlapping clinical features, such as fever, hepatosplenomegaly, and cytopenia, and thus the diagnosis is often delayed. Bone marrow aspiration revealing hemophagocytosis and the presence of *Leishmania amastigotes* is crucial for diagnosis [6,7]. Early recognition and treatment of VL-associated HLH is critical to improving outcomes. Liposomal amphotericin B is the first-line therapy for VL [6,7]. In some cases, additional immunosuppressive therapy may be required to control the hyperinflammatory response [7].

Here, we report a rare case of hemophagocytic lymphohistiocytosis secondary to visceral leishmaniasis.

## Case presentation

A 21-year-old female patient presented with a recent diagnosis of HIV infection, stage C3, with a CD4+ lymphocyte count of 50/mm^3^. Prior hospitalization in the context of severe *Pneumocystis jirovecii *pneumonia, she completed 21 days of antibiotic therapy with trimethoprim/sulfamethoxazole and adjuvant prednisone (40 mg orally twice daily for five days, followed by 40 mg orally once daily for five days, followed by 20 mg orally once daily for 11 days) with good response to the treatment. Antiretroviral therapy (ART) with tenofovir disoproxil fumarate (TDF)/emtricitabine (FTC) + dolutegravir (DTG) was started and she was discharged.

One week after starting ART, she presented with fever, erythematous rash on the upper limbs and trunk, choluria, and jaundice. No history of abdominal pain, nausea, or vomiting.

On physical examination, vital signs were stable: blood pressure 133/94 mmHg, elevated heart rate 125/min, SpO_2_ (room air) 98%, and a temperature of 38.8°C. She was alert, oriented, and cooperative and presented a maculopapular rash on the upper limbs, trunk, and abdomen. The chest was symmetric with no respiratory effort. Breath sounds were clear to auscultation bilaterally. Heart sounds were regular and no murmurs. The abdomen was flat, without tenderness or guarding. No edema was present.

Laboratory tests showed anemia (Hb 10g/dL), leukopenia (2300/uL), thrombocytopenia (65 000/uL) and slight cholestasis (aspartate aminotransferase {AST} 168 U/L; alanine aminotransferase {ALT} 269 U/L; gamma-glutamyl transferase {GGT} 293 U/L; alkaline phosphatase {ALP} 236 U/L; total bilirubin 3.30 mg/dL; C-reactive protein {CRP} 3.00 mg/dL). An abdominal ultrasound was performed to better evaluate the liver and bile duct showing normal intra and extrahepatic bile duct, gallbladder with thin walls, and no stones or focal hepatic lesions.

The patient was admitted with the diagnosis of toxic hepatitis and hematologic toxicity secondary to ART and cotrimoxazole, and both therapies were discontinued, switching prophylaxis to atovaquone.

**Figure 1:**
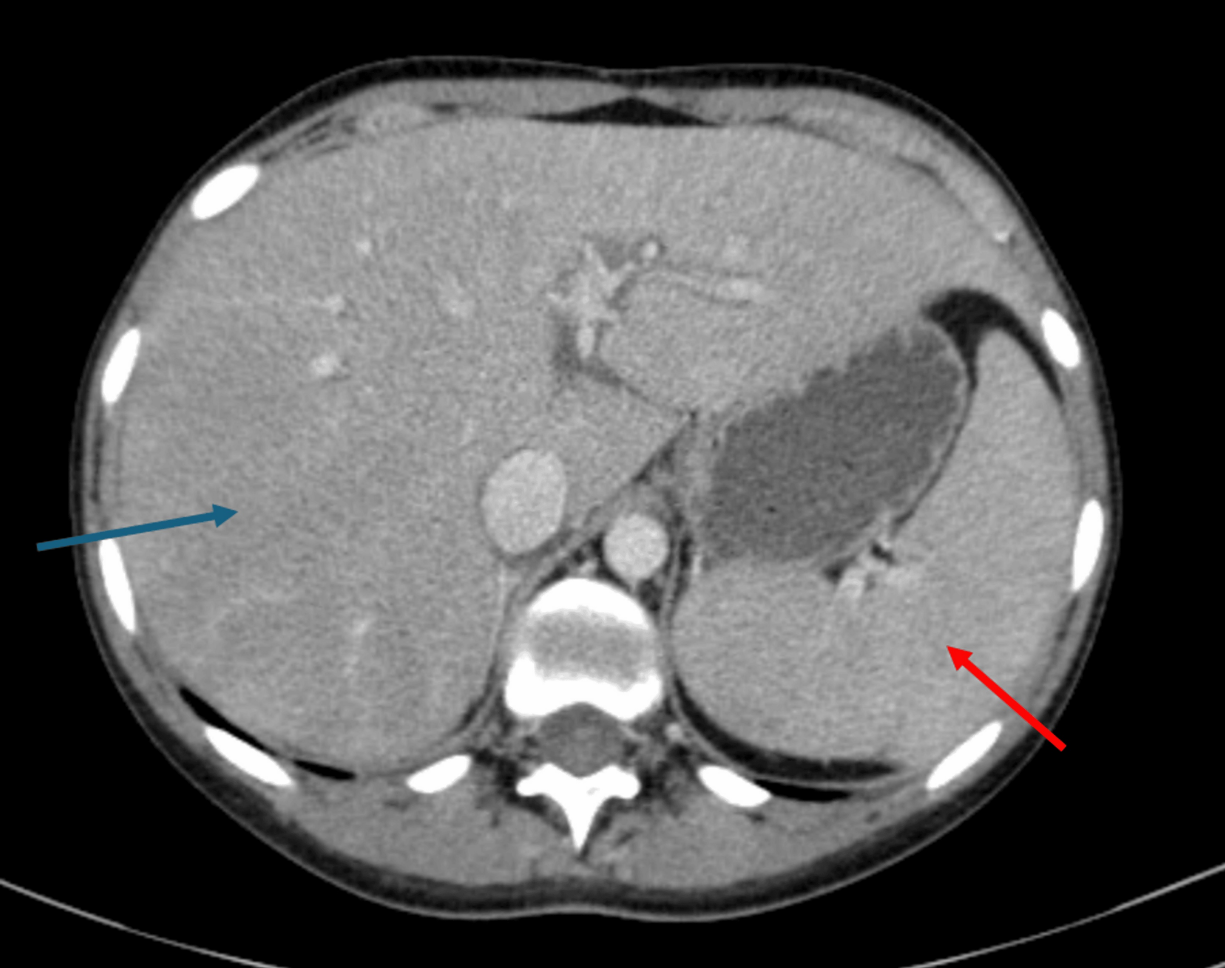
CT showing heterogenous hepatomegaly, 21.2 cm (blue arrow), and homogeneous splenomegaly, 13.3 cm (red arrow).

During hospitalization, she maintained a persistent fever, with a maximum of 39.4ºC. Both blood and urine cultures were collected and empirical antibiotic therapy with piperacillin tazobactam 4.5 g 6/6h was started. A thoracic-abdominal-pelvic CT scan was performed, showing only significant hepatomegaly (21.2 cm) and homogeneous splenomegaly (13.3 x 7.3 cm), which can be seen in Figure 1.

The patient maintained a fever despite antibiotic therapy, with negative blood and urine cultures. Worsening pancytopenia (hemoglobin 8.3 g/dL; leukocytes 850/µL; platelets 29,000/µL) was observed, and blood tests showed elevated ferritin (32,573 ng/mL) and triglycerides (658 mg/dL). Flow cytometry revealed low CD4+ (50/µL) and low NK cell activity.

**Figure 2:**
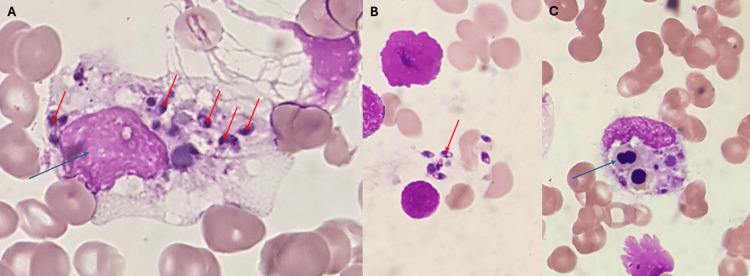
Bone marrow aspirate (Wright-Giemsa stain, 1000x). (A) Macrophage (blue arrow) engulfing leishmania (red arrow); (B) leishmania (red arrow); (C) monocyte engulfing leukocyte precursors (blue arrow).

Presenting multiple criteria for hemophagocytic lymphohistiocytosis, she was submitted to a bone marrow aspirate on day 6, with the results on day 7 showing *Leishmania amastigotes* and aspects of hemophagocytosis which can be seen in Figure 2.

The results of the laboratory analysis and microbiology are summarized in Table 1.

**Table 1:** Summary of clinical investigation. PCT: procalcitonin, EBV: Epstein-Barr virus, VZV: varicella-zoster virus, CMV: cytomegalovirus, ANAs: antinuclear antibodies, NK: natural killer (a type of immune cell).

Investigation	Value	Normal value (units)
Hemoglobin (minimum)	8.3 g/dL	12-16 g/dL
Leukocyte count (minimum)	850/uL	4000-11 000/uL
Platelet count (minimum)	29 000/uL	150 000-400 000/uL
Urea	17 mg/dL	<50 mg/dL
Creatinine	0.6 mg/dL	0.6-1.1 mg/dL
Aspartate aminotransferase (AST)	233 U/L	<35 U/L
Alanine aminotransferase (ALT)	102 U/L	<33 U/L
Gamma-glutamyl transferase (GGT)	191 U/L	7-32 U/L
Alkaline phosphatase (ALP)	233 U/L	35-105 U/L
Lactate dehydrogenase (LDH)	1503 U/L	135-214 U/L
Bilirubin, total	4.10 mg/dL	<1.2 mg/dL
Bilirubin, direct	3.90 mg/dL	<0.3 mg/dL
C-reactive protein (CRP)	6.90 mg/dL	<0.5 mg/dL
PCT	0.3 ng/mL	<0.5 ng/mL
Ferritin	32573.0 ng/mL	15-150 ng/mL
Triglycerides	658 mg/dL	<150 mg/dL
IgM EBV	Negative	
Herpes 1, 2, & VZV	Negative	
CMV	Negative	
ANAs	<1:160	
CD4+	50/uL	
NK	44/uL	90-590 cells/uL
Blood cultures	Negative	
Urine cultures	Negative	
Bone marrow aspirate	Leishmania and hemophagocytic cells	

The patient was diagnosed with HLH secondary to VL, she received treatment with liposomal amphotericin B 240 mg intravenous (4mg/Kg) on days 1-5 and then on days 10, 17, 24, and 31. ART was restarted with tenofovir alafenamide (TAF)/ FTC + darunavir/cobicistate (DRV/COBI) and she was discharged after completing the first five days of amphotericin. The rest of the regimen (doses on days 10, 17, 24, and 31) was completed in a day hospital setting without complications.

Re-evaluated in a follow-up appointment two months later, she was asymptomatic, with normalization of cell lineages (hemoglobin 12.2 g/dL; leukocytes 4420/uL; platelets 155,000/uL), ferritin 93 ng/mL, CD4+ 583 (22%). She maintained follow-up for HIV with Internal Medicine every six months.

## Discussion

HLH is associated with hyper-cytokinemia and organ infiltration by phagocytosing histiocytes that can lead to multi-organ dysfunction. The diagnostic framework for HLH, as established by the HLH-2004 criteria, involves a combination of clinical, laboratory, and histopathological findings, and typically the patients present with fever, cytopenias, organomegaly, coagulopathy, and lipid changes [1,4]. This syndrome can be categorized as either familial or sporadic, both being triggered by a variety of events. In addition to EBV infection, other viruses, bacteria, tuberculosis, malaria, and leishmaniasis have been reported to trigger HLH. Other triggers include inherited syndromes, malignancy, rheumatologic disorders, and drugs [1,8,9].

VL is caused by protozoan parasites of the leishmania species, is transmitted by infected sandflies, and though relatively rare in Portugal, continues to be a significant public health issue. VL is an uncommon but notable trigger for HLH, particularly in immunocompromised individuals.

The clinical manifestations of VL and HLH overlap significantly, including fever, hepatosplenomegaly, and cytopenias, often leading to diagnostic delays. Bone marrow aspiration (BMA), revealing hemophagocytosis and the presence of *Leishmania amastigotes*, is critical for diagnosis [6]. The initial BMA often fails to detect amastigote forms, hence, when there is a strong clinical suspicion of HLH associated with VL, it is essential to conduct multiple BMAs and have them reviewed by experienced physicians. Spleen aspiration could be an alternative; however, it is associated with higher risk, such as life-threatening hemorrhages, and, therefore is typically avoided unless absolutely necessary [7,8].

When HLH is triggered by an acute infection, treating the underlying infection is appropriate as it may eliminate the stimulus for immune activation. Stable patients might be managed with just the treatment of the triggering condition without needing HLH-specific therapy. However, for clinically unstable patients, the primary goal is to suppress life-threatening inflammation. This is achieved through induction therapy with dexamethasone and etoposide [4].

The treatment for VL primarily involves liposomal amphotericin B (L-AmB), which is associated with the highest therapeutic efficacy and the most favorable safety profile. For patients who cannot tolerate L-AmB or in places where it is not available, alternatives include miltefosine, pentavalent antimonial drugs, or paromomycin [7]. 

The presented case involves an immunocompromised, 21-year-old female patient, who had sustained fever, jaundice, and maculopapular rash, along with significant hematologic abnormalities, initially suggested drug-induced toxicity. However, the progressive worsening of her pancytopenia and the lack of response to empirical antibiotic therapy prompted further investigation. The identification of *Leishmania amastigotes* in bone marrow aspirate confirmed the diagnosis of VL, and the presence of multiple HLH diagnostic criteria (fever, cytopenias, hypertriglyceridemia, elevated ferritin, organomegaly, low NK activity, and hemophagocytosis) established the diagnosis of HLH.

The treatment regimen for this patient involved liposomal amphotericin B, which is the first-line therapy for VL. This therapeutic approach, combined with the management of her HIV infection, led to a marked clinical improvement.

In this case, it was possible to establish the presence of leishmania in the first bone marrow aspirate (BMA), unlike most cases, as shown in a case series and literature review published in 2022 in which BMA was negative for leishmania in 59.2% of cases [7]. Also, it is important to mention that the diagnosis was made in a timely manner, seven days after admission. In the same case series, there was a mean delay in diagnosis of the reported cases of 13 weeks [7]. This is partially justified by high clinical suspicion that prompts a timely BMA with a diagnosis the following day. 

The patient’s response underscores the importance of prompt diagnosis and appropriate treatment for VL-associated HLH to mitigate the severe consequences of this hyperinflammatory syndrome.

## Conclusions

This case illustrates the rare but serious complication of HLH secondary to VL in an immunocompromised patient. It emphasizes the need for awareness of this potential diagnosis in similar clinical scenarios and the importance of a comprehensive diagnostic and therapeutic approach to improve patient outcomes. The successful resolution of the patient’s condition following targeted therapy for VL and appropriate management of her HIV infection demonstrates the efficacy of a well-coordinated treatment strategy in managing complex cases of HLH.
